# Gestation and breastfeeding in schistosomotic mice differentially alters the expression of histone deacetylases (HDACs) in adult offspring

**DOI:** 10.1590/0074-02760190366

**Published:** 2020-02-03

**Authors:** Gabriela Calixto Ribeiro de Holanda, Fabrício Oliveira Souto, Maria da Conceição Silva, Virgínia Maria Barros de Lorena, Vlaudia Maria Assis Costa, Monica Camelo Pessôa de Azevedo Albuquerque, Valdênia Maria Oliveira de Souza, José Luiz de Lima

**Affiliations:** 1 Universidade Federal de Pernambuco, Laboratório de Imunopatologia Keizo Asami, Recife, PE, Brasil; 2 Fundação Oswaldo Cruz-Fiocruz, Instituto de Pesquisas Aggeu Magalhães, Recife, PE, Brasil

**Keywords:** breastfeeding, epigenomics, histone deacetylases, pregnancy, schistosomiasis

## Abstract

**BACKGROUND:**

Breastfeeding or gestation in schistosomotic mothers can cause long-term alterations in the immune response of offspring.

**OBJECTIVES:**

Evaluate the expression of histone deacetylases (HDACs) (all classes), the production of cytokines by T and B lymphocytes and macrophages, and the frequency of CD4^+^CD25^+^FoxP3^+^-cells in adult offspring born and/or suckled by schistosomotic mothers.

**METHODS:**

We harvested splenocytes from offspring born to (BIM), suckled by (SIM), or born to/suckled by (BSIM) schistosomotic mothers and animals from noninfected mothers (Control) at seven-weeks old and cultured them with/without Concanavalin A. HDAC expression was evaluated by real-time quantitative polymerase chain reaction (qPCR), and cytokines and membrane markers were evaluated by fluorescence-activated cell sorting (FACS).

**FINDINGS:**

Compared to Control, BIM mice showed increased expression of HDAC9 and frequency of CD4^+^IL-10^+^-cells. The SIM group had increased expression of HDAC1, HDAC2, HDAC6, HDAC7, HDAC10, Sirt2, Sirt5, Sirt6, and Sirt7. The BSIM group only had increased HDAC10 expression. The SIM and BSIM groups exhibited decreased frequencies of CD4^+^IL-4^+^-cells and CD4^+^CD25^+^FoxP3^+^-cells, along with a higher frequency of CD14^+^IL-10^+^-cells and an increase in CD45R/B220^+^IL-10^+^-cells. The BSIM group also showed a high frequency of CD4^+^IL10^+^-cells.

**MAIN CONCLUSIONS:**

Breastfeeding induced the expression of HDACs from various classes involved in reducing inflammatory responses. However, gestation enhanced the expression of a single HDAC and breastfeeding or gestation appears to favour multiple IL-10-dependent pathways, but not cells with a regulatory phenotype.

A high prevalence of chronic schistosomiasis in pregnant women and women of childbearing age has been reported,^1^ and the effects of maternal infection have raised questions regarding the immunity of the offspring.

It is known that the immunological status of schistosomotic mothers can induce long-term alterations in the immune response of the offspring.^2^ ^,^ ^3^ ^,^ ^4^ ^,^ ^5^ ^)^ An experimental study on the effects of gestation and breastfeeding in infected mothers, separately, showed that gestation in these mothers led to potential immunosuppression in adult offspring, with elevated production of IL-10 and lower levels of anti-ovalbumin (OA) antibodies.^2^ In addition, offspring born to infected mothers had a lower frequency of B lymphocytes, and the capacity for antigen presentation by CD11c+ cells was partially impaired.^3^ In contrast, it has also been observed that adult mice previously breastfed by schistosomotic mothers exhibited improvement in the production of anti-OA antibodies^2^ and in the antigen presentation ability of B lymphocytes through an increase in surface frequency of CD40+/CD80+ in these cells.^3^ However, whether these alterations in the immune response of adult offspring from infected mothers are due to epigenetic changes from the perinatal period remains unclear.

Studies have correlated post-transcriptional changes in the chromatin, through histone acetylation/deacetylation, with the immune response.^6^ ^,^ ^7^ ^,^ ^8^ In an experimental study on antigen presenting cells (APCs), it was demonstrated that histone deacetylase (HDAC)6 is required for transcriptional activation of IL-10 gene expression in macrophages and dendritic cells through activation of STAT3.^6^ Another study using pancreatic beta cell lines showed that knockdown of HDAC1 increased IFN-γ-induced STAT1 phosphorylation.^7^ Kosciuczuk et al.^8^ showed that deacetylation of cyclin-dependent kinase 9 induced by Sirtuin 2 promotes STAT1 phosphorylation during type I interferon responses.

In addition, it has been demonstrated that the role of epigenetic markers can be remodelled during the perinatal period, and may trigger lasting influences on the epigenome of the offspring.^9^ Mice prenatally administered with *Acinetobacter lwoffii* F78 displayed increased acetylation of histone H4 in the interferon (IFN)-γ gene in their offspring, and conferred protection against asthma after challenge with OA, which is associated with positive regulation of IFN-γ production.^10^ Song et al.^11^ found that offspring from mothers with peanut allergy had elevated IgE-specific levels, high levels of histamine and resultant increased production of Th2 cytokines, and reduction of DNA methylation at CpG sites of the IL-4 gene promoter after sensitisation.

Histone acetylation is the most commonly studied epigenomic alteration, for stimulation of transcription, and in turn, is reversibly regulated by the balance between the activity of histone acetyltransferases (HATs) and HDACs.^12^ HDACs have been classified as class I (HDAC1, HDAC2, HDAC3 and HDAC8), class IIa (HDAC4, HDAC5, HDAC7, and HDAC9), class IIb (HDAC6 and HDAC10), class III (SIRT1 to SIRT7), and class IV (HDAC11)^13^ and are increasingly studied due to their interference in the pathways of mechanisms associated with the pathogenesis of various cancers and other inflammatory diseases.^14^ ^,^ ^15^ ^,^ ^16^

Although research that relates epigenetic alterations to the maternal-foetal relationship can be found, there are no studies that report the effects of gestation and/or breastfeeding on the expression of HDACs, and the implications for the immune system of offspring from schistosomotic mothers. To investigate, we have evaluated whether the expression of enzymes involved in chromatin remodelling through histone deacetylation can be altered due to gestation or breastfeeding from *Schistosoma mansoni*-infected mothers. Our results could aid in the discovery of therapeutic targets that improve the immunity of individuals who previously contacted immunological factors resulting from infection during perinatal period.

## MATERIALS AND METHODS

*Animals and maternal infection* - Four-week-old Swiss Webster female mice were infected subcutaneously (s.c.) with 20 *S. mansoni* cercariae, strain São Lourenço da Mata (SLM). On the 45th day, infection was confirmed by the Kato-Katz method.^17^ On the 60th day post-infection (dpi), oestrus was synchronised among mice via the administration of 5 i.u. (100 µL) of equine chorionic gonadotrophin hormone, followed by an additional injection with 5 i.u. (100 µL) of human chorionic gonadotrophin 48 h later. Females were housed with male mice at a 1:1 ratio, and successful mating was confirmed by presence of a vaginal plug. The same procedure was performed in noninfected females, and seven-week-old male offspring were taken for the experimental and control groups. The mice were housed in the animal care facility at the Aggeu Magalhães Institute (IAM), Oswaldo Cruz Foundation (Fiocruz), municipality of Recife, State of Pernambuco, Brazil.

*Adoptive nursing and study groups* - Immediately after birth, new-born mice from *S. mansoni-*infected or noninfected mothers were rehoused with mothers from the opposite group. After adoptive nursing, offspring born from infected mothers (BIM) were suckled by noninfected mothers, and offspring from noninfected mothers were suckled by infected mothers (SIM). A separate group was born from and suckled by schistosomotic mothers (BSIM). Animals born from noninfected females were suckled by their mothers (Control).

*Cell culture* - Spleens from each animal (seven-weeks-old) were harvested after euthanasia by cervical dislocation. Cell suspensions were prepared in RPMI-1640 (Sigma-Aldrich, St. Louis, USA) supplemented with HEPES (10 µM), 2-mercaptoethanol (0.05 µM), 216 mg of L-glutamine/L, gentamicin (50 mg/L), and 5% of foetal bovine serum (FBS) (Sigma-Aldrich, St. Louis, USA). Cells from each group (n = 8-10) were cultivated at a final concentration of 2 × 10^7^ cells/mL in tissue culture plates (Costar Culture Plates, City, USA) and stimulated with concanavalin-A (Con-A) (5 μg/mL), or without antigenic stimulus (Basal), at 37ºC in 5% CO_2_. Cultured cells were harvested after 24 h and assayed for immunophenotyping and real-time quantitative polymerase chain reaction (qPCR).

**Fig. 1:**
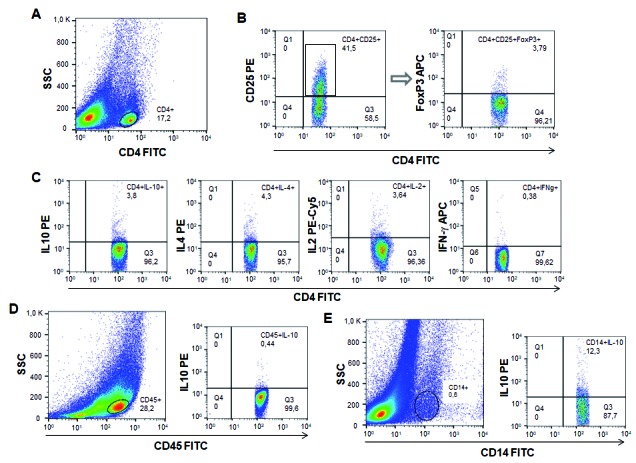
representation of the gating strategy used to analyse the different subpopulations of cells. FL1/anti-CD4-FITC versus laser side-scatter (SSC) dot plot (A), CD4+CD25+FoxP3+ cells dot plots (B), cytokine producing T CD4+ lymphocytes dot plots (C), IL-10 producing CD45R/B220+ cells dot plot (D) and IL-10 producing CD14+ cells dot plot (E).

*Flow cytometry analyses* - 5 μL of Golgi Stop (per 2 × 10^7^ cells) were added to each well containing splenic cells under different stimuli, then the cells were vortexed and returned to the CO_2_ incubator at 37ºC for four additional hours. Spleen cells were subjected to double-labelling with fluorochrome-labelled antibody solutions at a concentration of 0.5 mg/10^6^ cells: FITC anti-mouse CD4, and PE anti-mouse IL-4, APC anti-mouse IFN-γ, PE anti-mouse IL-10, or PerCP-Cy-5.5 anti-mouse IL-2; FITC anti-mouse CD4, PE anti-mouse CD25, and APC anti-mouse FoxP3; FITC anti-mouse CD45R (B220) or FITC anti-mouse CD14, and PE anti-mouse IL-10 (BD Biosciences Pharmingen). After staining, preparations were washed with phosphate-buffered saline (PBS) containing azide (0.1%) and FBS (3%). After centrifugation, the cell pellet was resuspended in PBS with paraformaldehyde (0.5%) and maintained at 4ºC until data acquisition, which was performed using a FACSCalibur (BD-Pharmingen, New Jersey, USA) flow cytometer and acquisition of a minimum 50,000 lymphocytes or 5,000 monocytes. The frequency of positive cells was analysed using FlowJo software, with quadrant gating set based on negative populations and isotype controls. A descriptive analysis of the frequency of cells in the upper right quadrant (double-positive cells) was performed. Distinct gating strategies were used to analyse each subpopulation of cells (Fig. 1). T lymphocyte subpopulations were first selected as CD4 high cells on FL1/anti-CD4-FITC versus laser side-scatter (SSC) dot plots (Fig. 1A). Following this gating procedure, a second gate was set using FL1/anti-CD4-FITC versus FL2/anti-CD25-PE; then, a third gate was established to generate representative 2-dimensional graphics using FL1/anti-CD4-FITC versus FL4/anti-FoxP3-APC to identify triple staining for CD4+CD25+FoxP3+ (Fig. 1B). The frequency of cytokine-expressing cells was further determined on FL1/anti-CD4-FITC versus FL2/anti-IL10-PE or anti-IL4-PE, FL3/anti-IL2-Percp-Cy-5.5 or FL4/anti-IFN-γ-APC dot plots by quadrant statistic measurements, and expressed as percentage of cytokine T CD4+ lymphocyte (Fig. 1C). B Cells and monocytes were first selected on CD45-high or CD14-high cells using FL1/anti-CD45 or CD14-FITC versus SSC dot plots, and the frequency of IL-10 producing cells was subsequently determined using FL1/CD45-FITC or FL1/CD14-FITC versus anti-IL10-PE dot plots and quadrant statistic measurements (Fig. 1D-E).

The results are expressed as the median frequency of cells from each group ± standard error.

*qPCR analysis* - Total RNA from splenic cells was extracted using the ReliaPrep^TM^ RNA Cell Miniprep System Kit (Promega, Madison, WI) according to the manufacturer’s instructions. Complementary DNA (cDNA) was generated with the QuantiTect Reverse Transcription Kit (Qiagen, Hilden, Germany). Quantitative PCR was performed using SYBR Green master mix (Applied Biosystems, Foster City, CA, USA) on the 7500 Real Time System (Applied Biosystems Foster City, CA, USA) machine. Results were normalised to the housekeeping gene β-Actin. Relative expression levels were calculated using 2^ΔΔCt^. Primers were designed using Primer3Plus software, and the sequences are described in Table.

**TABLE:** Primers used in quantitative real-time polymerase chain reaction (qPCR)

Target gene (Accession number)	Sequence 5’-3’ (Forward and reverse)	Tm	CG %	Amplicon length (bp)
HDAC1 (NM_008228.2)	CCGGTTAGGTTGCTTCAATC	59.6	50	118
	AACATTCCGGATGGTGTAGC	59.8	50	
HDAC2 (NM_008229.2)	TATTATGGCCAGGGTCATCC	59.6	50	119
	TCAGCAGTGGCTTTATGAGG	59	50	
HDAC3 (NM_010411.2)	ATGCAGGGTTTCACCAAGAG	60.1	50	117
	TGTTGCTCCTTGCAGAGATG	60.1	50	
HDAC4 (NM_207225.2)	CCGCCAGCAGTTTTAAAGTC	59.9	50	92
	ACCGAATGGAGATGCTCAAC	60.1	50	
HDAC5 (NM_001077696.1)	ACTTTCCCCTCCGTAAAACG	60.3	50	116
	AACAGTGCCATCCTTTCGAC	60.1	50	
HDAC6 (NM_010413.3)	ATCTCAGCTGGCTTTGATGC	60.5	50	116
	ATAATACGGCCACCAGCAAG	60.0	50	
HDAC7 (NM_001204275.1)	ATGATGGCCTGGAACATAGG	59.8	50	75
	GATGCTGCTGCAGAGAAATG	59.7	50	
HDAC8 (NM_027382.4)	AGGGAATCTGAAGCATGTGG	60.1	50	131
	CAAATTTCCCCTGCAGTCAC	60.5	50	
HDAC9 (NM_001271386.1)	TTTGAGGTGGCAGAATCCTC	60.2	50	106
	GAGCTGAAGCCTCATTTTCG	60.1	50	
HDAC10 (NM_199198.2)	AACAGGAGCTGTGCACAATG	59.9	50	143
	TCCTCTGCAGCCCATATTTC	60.2	50	
HDAC11 (NM_144919.2)	TGATGGGGTTGAACACTGAG	59.5	50	128
	AGCAGCCCCTTAAAAACTCC	59.7	50	
Sirt1 (NM_019812.3)	GCCCTCAATTTCTGTTCTGC	59.8	50	150
	TTTTGAGTGCTCCAGACACG	60.0	50	
Sirt2 (NM_019812.3)	ACGGCTGCTCATTAACAAGG	60.3	50	88
	GTCAAAATCCATGCCACCTC	60.3	50	
Sirt3 (NM_001177804.1)	CATATGGGCTGATGTGATGG	59.8	50	141
	AGATCTGCCAAAGCGAAGTC	59.6	50	
Sirt4 (NM_001167691.1)	CGAGCAAAAGCTCCCAATAG	60.0	50	145
	TTCCAGCCTTTGGACATCAG	61.2	50	
Sirt5 (NM_178848.3)	CCAGCTTTAGCAGGAAAAGG	59.1	50	139
	CCAGGTTTTCTCCAAACCAC	59.4	50	
Sirt6 (NM_181586.3)	TGTCCAACACAGCTCCTTTC	58.9	50	97
	CTTCCACATGTGTGGGATTC	58.8	50	
Sirt7 (NM_001363439.1)	AGCTTCGGGATACCATTGTG	60	50	104
	CAGGATTGTGTCTGCTTTGC	59.4	50	
β-Actin (NM_007393.5)	TTGCTGACAGGATGCAGAAG	61.1	50	147
	TGATCCACATCTGCTGGAAG	59.8	50	

bp: base pair; CG: cytosine and guanine content; HDAC: histone deacetylase 1-11; Sirt: Sirtuin 1-7; Tm (melting temperature) was calculated at default settings of 0.25 µM oligo concentration and 50 µM Na. Primers were designed using Primer3Plus software (http://www.bioinformatics.nl/cgi-bin/primer3plus/primer3plus.cgi).

*Statistical analysis* - Results were subjected to Barlett’s test to verify whether the distribution of the data was normal. After verifying that the results did not follow a normal distribution, the Kruskal-Wallis test was used, followed by Dunn’s multiple comparison test when statistical significance was shown. For statistical analysis, we used GraphPad Prism v.5.0 (GraphPad Software, San Diego, CA, USA) and findings were considered significant at p < 0.05. All procedures were performed in triplicate to evaluate reproducibility, and images refer to one representative of at least three independent studies.

*Ethics* - The animal protocol was approved by the Ethical Commission on Animal Use of the Fiocruz (113/2017) and is in accordance with the Ethical Principles in Animal Research adopted by the Brazilian College of Animal Experimentation.

## RESULTS

**Fig. 2:**
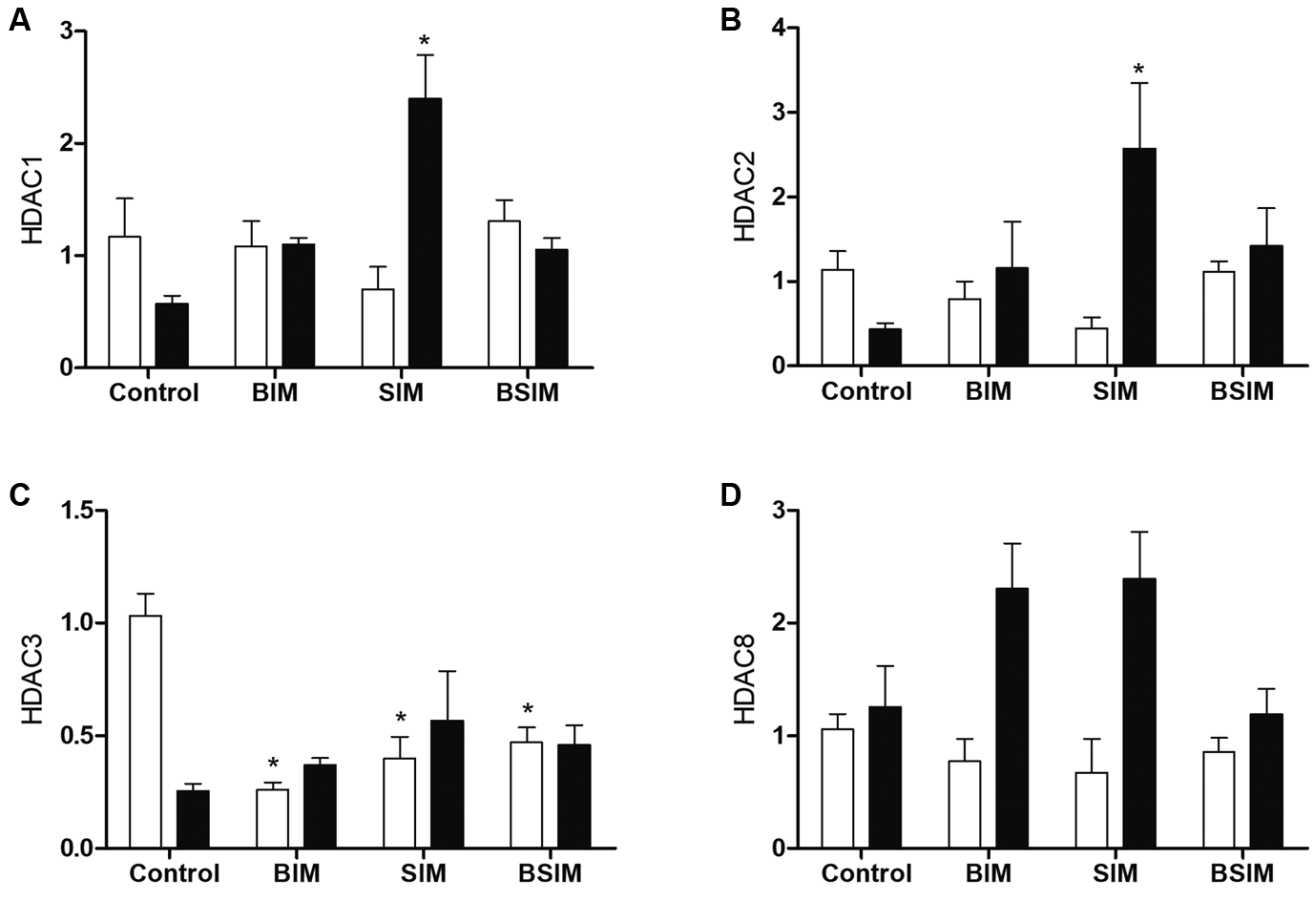
relative expression of class I histone deacetylases (HDACs). HDAC1 (A), HDAC2 (B), HDAC3 (C) and HDAC8 (D) in splenocytes from *Swiss Webster* mice (seven weeks) born (BIM), breastfed (SIM), or born and breastfed (BSIM) in infected mothers, or uninfected mothers (Control), cultured for 24 h in the presence of mitogen (Concanavalin A) (5 μg/mL, black bar) or with culture medium (BASAL, white bar). The relative expression was verified by real-time quantitative polymerase chain reaction (qPCR). The results represent the median and standard error for 8-10 animals/group. ^*^p < 0.05 compared to control group.

*Relative expression of HDAC in animals born and/or breastfed from schistosomotic mothers* - To verify the epigenetic profile of the animals born and/or breastfed from schistosomotic mothers, real time qPCR was performed on spleen cells cultured for 24 h in absence (basal) or presence of mitogenic stimulus (ConA). For class I HDAC, the basal relative expressions of HDAC1 and HDAC2 were not altered, but with mitogenic stimulus, the group of animals which were breastfed only (SIM) exhibited increased expression compared to the Control (Fig. 2A-B). The groups BIM, SIM, and BSIM had decreased basal relative expression of HDAC3, but with mitogenic stimulus the relative expression was similar to Control (Fig. 2C). There was no difference in the relative expression of HDAC8 either at baseline or with mitogenic stimulation (Fig. 2D).

**Fig. 3:**
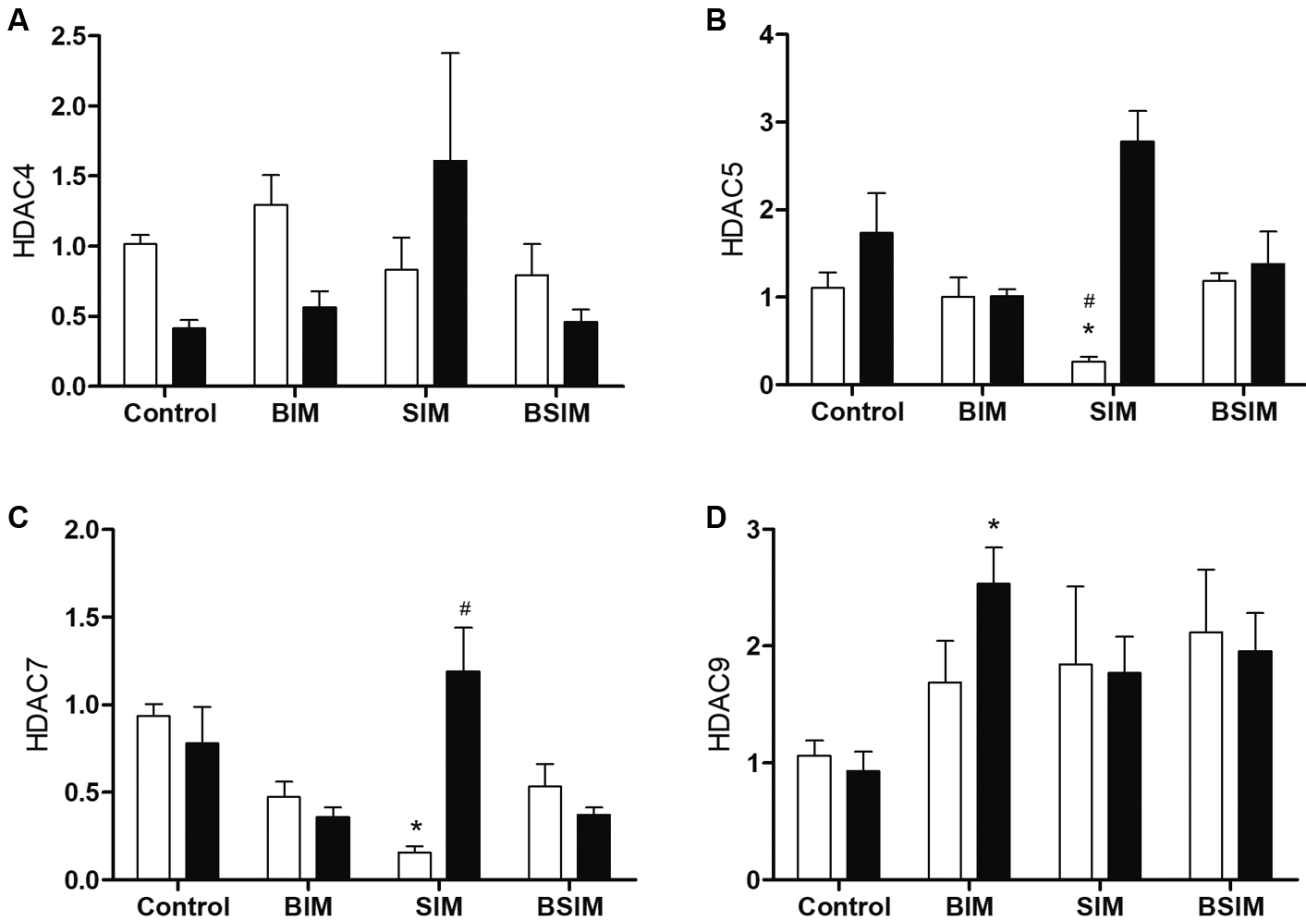
relative expression of class IIa histone deacetylases (HDACs). HDAC4 (A), HDAC5 (B), HDAC7 (C) and HDAC9 (D) in splenocytes from *Swiss Webster* mice (seven weeks) born (BIM), breastfed (SIM), or born and breastfed (BSIM) in infected mothers, or uninfected mothers (Control), cultured for 24 h in the presence of mitogen (Concanavalin A) (5 μg/mL, black bar) or with culture medium (BASAL, white bar). The relative expression was verified by real-time quantitative polymerase chain reaction (qPCR). The results represent the median and standard error for 8-10 animals/group. ^*^p < 0.05 compared to control group. ^#^p < 0.05 compared to BIM and BSIM groups.

Class IIa HDACs were analysed, and we saw that there was no difference in HDAC4 expression (Fig. 3A). The relative expression of HDAC5 in the SIM group was lower compared to Control, BIM and BSIM at baseline, but there was no difference among the groups under mitogenic stimulus (Fig. 3B). For HDAC7 the SIM group had lower expression than Control at baseline, while under mitogenic stimulus the SIM group had increased expression compared to the BIM and BSIM groups (Fig. 3C). Regarding HDAC9, there was no difference at baseline, but the BIM group showed a higher relative expression compared to Control under mitogenic stimulus (Fig. 3D).

**Fig. 4:**
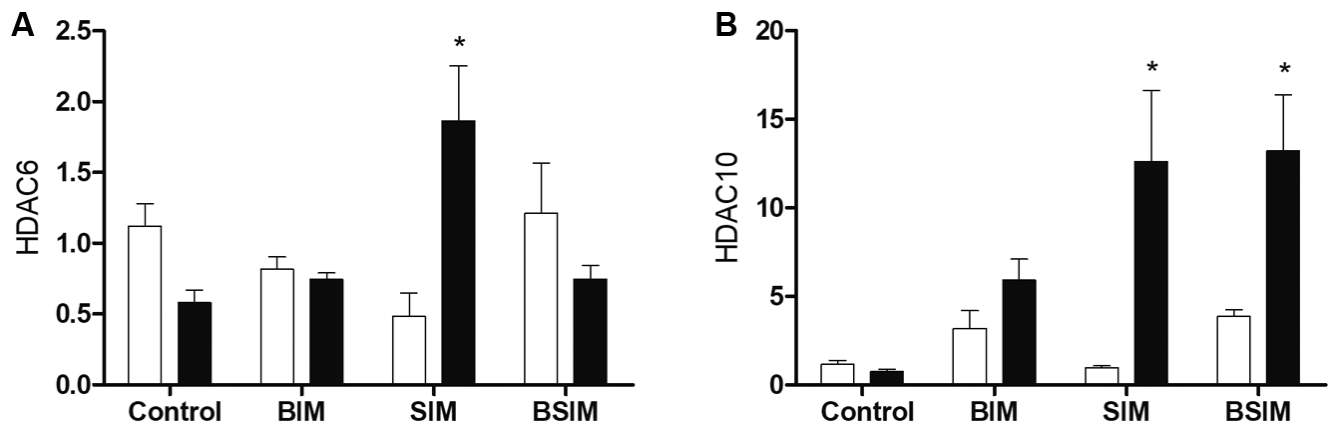
relative expression of class IIb histone deacetylases (HDACs). HDAC6 (A) and HDAC10 (B) in splenocytes from *Swiss Webster* mice (seven weeks) born (BIM), breastfed (SIM), or born and breastfed in (BSIM) infected mothers, or uninfected mothers (Control), cultured for 24 h in the presence of mitogen (Concanavalin A) (5 μg/mL, black bar) or with culture medium (BASAL, white bar). The relative expression was verified by real-time quantitative polymerase chain reaction (qPCR). The results represent the median and standard error for 8-10 animals/group. ^*^p < 0.05 compared to control group.

Regarding HDACs 6 and 10 (class IIb), there was similar expression among all groups, and did not differ significantly from Control. However, in response to mitogenic stimulus, the expression of HDAC6 and HDAC10 was increased in the SIM group, and HDAC10 was increased in the BSIM group (Fig. 4A-B).

**Fig. 5:**
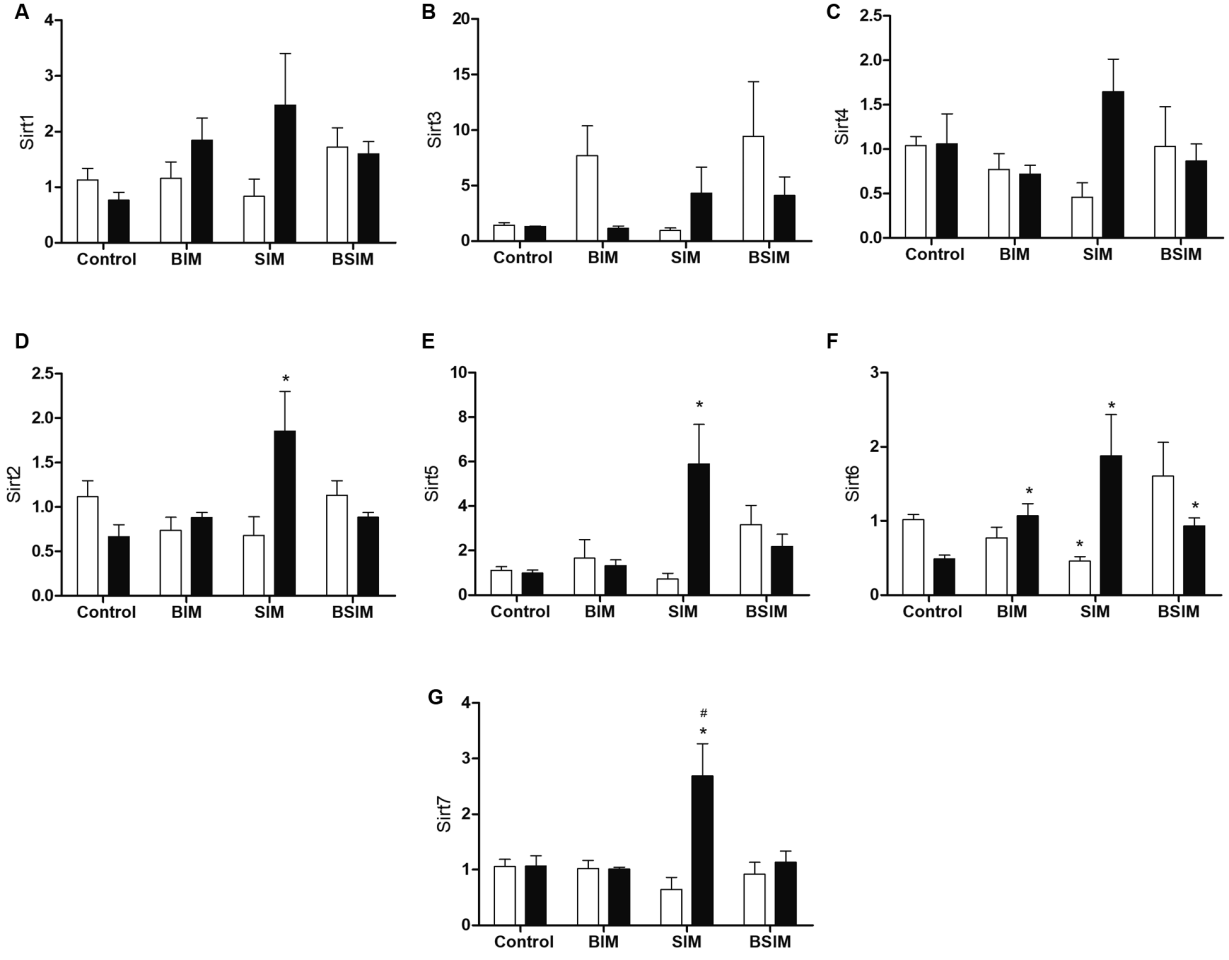
relative expression of class III histone deacetylases (HDACs). Sirt1 (A), Sirt3 (B), Sirt4 (C), Sirt2 (D), Sirt5 (E), Sirt6 (F) and Sirt7 (G) in splenocytes from *Swiss Webster* mice (seven weeks) born (BIM), breastfed (SIM) or born and breastfed (BSIM) in infected mothers, or uninfected mothers (Control), cultured for 24 h in the presence of mitogen (Concanavalin A) (5 μg/mL, black bar) or with culture medium (BASAL, white bar). The relative expression was verified by real-time quantitative polymerase chain reaction (qPCR). The results represent the median and standard error for 8-10 animals/group. ^*^p < 0.05 compared to control group. ^#^p < 0.05 compared to BIM and BSIM groups.

Among sirtuins (class III), the expressions of Sirt1, Sirt3, and Sirt4 were found to be similar among all groups under the culture conditions used (Fig. 5A-C). Sirt2 and Sirt5 did not show any differences in the basal groups, but increased expression was observed in the SIM group compared to Control under mitogenic stimulation (Fig. 5D-E). Fig. 5F shows that compared to Control, the expression of Sirt6 in the SIM group was lower at baseline. However, under mitogenic stimulus, there was a significant increase in the expression of Sirt6 in all experimental groups (BIM, SIM, and BSIM). Although there was no baseline difference for Sirt7, the SIM group had increased expression with mitogenic stimulus compared to the Control, BIM, and BSIM groups (Fig. 5G).

**Fig. 6:**
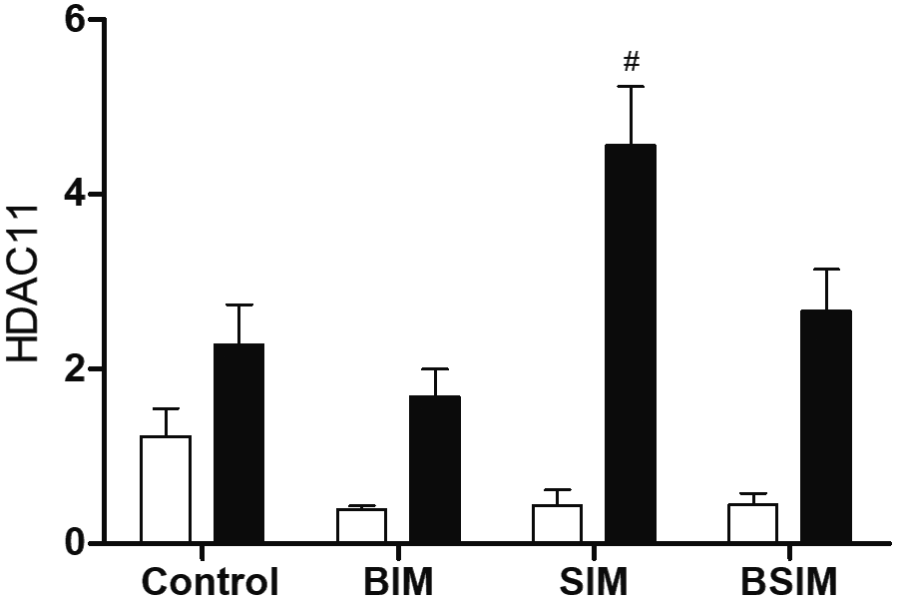
relative expression of class IV histone deacetylases (HDACs). HDAC11 in splenocytes from *Swiss Webster* mice (seven weeks) born (BIM), breastfed (SIM) or born and breastfed(BSIM) in infected mothers, or uninfected mothers (Control), cultured for 24 h in the presence of mitogen (Concanavalin A) (5 μg/mL, black bar) or with culture medium (BASAL, white bar). The relative expression was verified by real-time quantitative polymerase chain reaction (qPCR). The results represent the median and standard error for 8-10 animals/group.

Class IV is composed only of HDAC11 which in this study showed no differences compared to Control, but the SIM group had higher relative expression compared to BIM under mitogenic stimulus (Fig. 6).

**Fig. 7:**
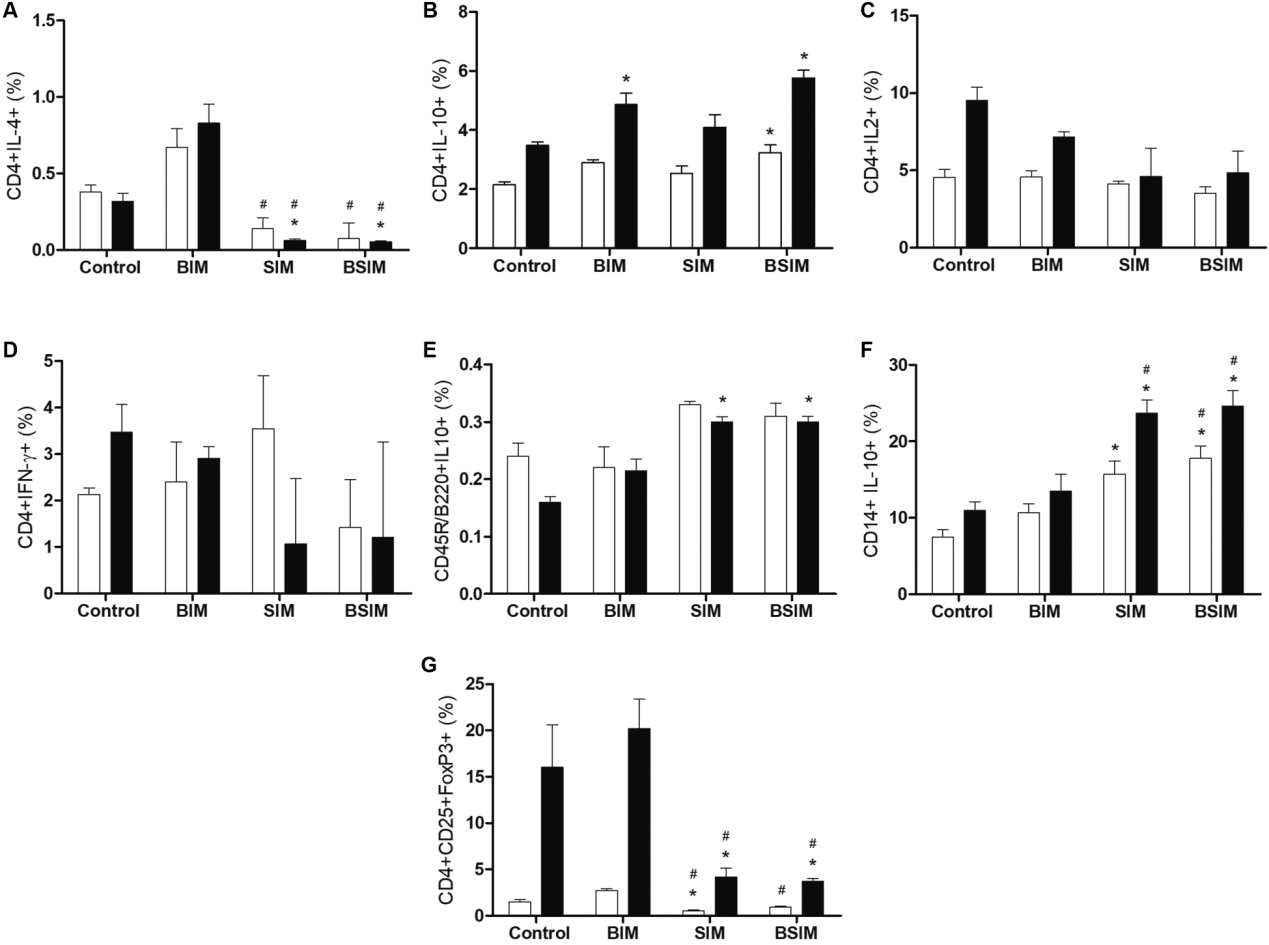
cytokine production by T and B lymphocytes and monocytes, and splenocytes expressing CD4+ CD25+ FoxP3+. Frequency of CD4+IL-4+ (A), CD4+IL-10+ (B), CD4+IL-2+ (C), CD4+IFN-γ+ (D), CD45R/B220+IL-10+ (E), CD14+IL-10+ (F) and CD4+CD25+FoxP3+ (G) in splenocytes from *Swiss Webster* mice (seven weeks) born (BIM), breastfed (SIM) or born and breastfed (BSIM) in infected mothers, or uninfected mothers (Control), cultured for 24 h in the presence of mitogen (Concanavalin A) (5 μg/mL, black bar) or with culture medium (BASAL, white bar). The frequencies were verified by flow cytometry. The results represent the median and standard error for 8-10 animals/group. ^*^p < 0.05 compared to control group. ^#^p < 0.05 compared to BIM and BSIM groups.

*Intracellular cytokines in T and B lymphocytes, and monocytes, and the frequency of regulatory T lymphocytes in animals born and/or breastfed* - Cytokine producing T lymphocytes were observed by labelling with CD4+/IL-4+, CD4+/IFN-γ+, CD4+/IL-10+, or CD4+/IL-2+, while B lymphocytes and monocytes were labelled with anti-CD45R/B220+ and anti-CD14+ antibodies, respectively, together with anti-IL-10+. T lymphocytes with a regulatory profile were evaluated by triple labelling CD4+CD25+FoxP3+. Frequencies were evaluated with mitogenic stimulus (ConA) and without (basal). Compared to the Control group, the frequency of CD4+/IL-4+ cells under basal conditions and mitogenic stimulation was lower in the SIM and BSIM groups (Fig. 7A). IL-10 production by CD4+ cells was higher in the BIM and BSIM groups under mitogenic stimulation (Fig. 7B). There were no differences among groups when the frequencies of CD4+IL-2+ and CD4+/IFN-γ+ cells (Fig. 7C-D) were analysed.

Regarding IL-10 production by B lymphocytes (Fig. 7E), it was slightly higher in the SIM and BSIM groups in response to mitogen. The SIM and BSIM groups also had an increased frequency of CD14+IL-10+ in comparison to the Control and BIM groups both at baseline and in response to mitogen (Fig. 7F).

For cells expressing CD4+CD25+FoxP3+, the SIM and BSIM groups exhibited decreased frequency relative to the Control and BIM groups, with and without mitogenic stimulus (Fig. 7G).

## DISCUSSION

Maternal infection by *S. mansoni* can alter the degree of immune competence of the offspring in the long term, either through in-utero contact or breastfeeding.^2^ ^,^ ^3^ ^,^ ^4^ ^,^ ^5^ In this study, the effects of gestation and breastfeeding were evaluated separately in *S. mansoni*-infected mothers. Therefore, the expression of HDACs, and cytokine production by lymphocytes and macrophages was assessed. These experiments were conducted using an *in vitro* system with broadly activated splenic cells, achieved using mitogenic stimulation.

Our findings show that breastfeeding from infected mothers induced the expression of HDACs from different classes which are involved in reducing the inflammatory response; however, gestation enhanced the expression of a single HDAC. These enzymatic changes induced by breastfeeding or gestation appear to inhibit cells with a regulatory phenotype (CD4+CD25+FoxP3+), but favour an IL-10-dependent pathway.

Only offspring generated from infected mothers showed increased expression of HDAC9. According to Tao et al.^18^, HDAC9 is linked to decreased generation and performance of regulatory T cells. It has been observed that animals genetically deficient in HDAC9 had increased mRNA expression of Foxp3, CTLA-4, and GITR and increased CD4+FoxP3+ cells in lymphoid tissues. Regarding the cell phenotypes studied here, we observed that the generated offspring had a higher frequency of IL-10 producing CD4+ T cells. Together, these data suggest that immunomodulation in utero induced by maternal infection does not favour the generation of cells with a regulatory phenotype, but does favour IL-10 production via HDAC9 in offspring. In studies using immunisation with ovalbumin as adjuvant, gestation in infected mothers was correlated with increased production of IL-10.^2^ ^,^ ^5^Furthermore, Li et al.^19^ reported the positive regulation of HDAC9 in immature peritoneal macrophages through the action of acetyltransferase Dnmt3, leading to increased production of IFN-α and IFN-β via deacetylation of kinase TBK1. Mice deficient for Dnmt3a showed greater susceptibility to infection by vesicular stomatitis virus (VSV). Thus, it is possible that gestation in schistosomotic mothers could alter the innate immune response against viral infections in offspring.

Prior exposure to the breast milk of schistosomotic mothers positively altered the expression of HDACs from different classes linked to reduction and/or resolution of the inflammatory response.^8^ ^,^ ^20^ ^,^ ^21^ There was an increase in the relative expression of HDACs 1, 2 and 7, all of which show anti-inflammatory activity by inhibiting the transcription of NF-κβ and decreasing the production of inflammatory cytokines (TNF-α, IL-1, IL-6).^7^ ^,^ ^22^ ^,^ ^23^ There was a decrease in HDAC3 expression in all experimental groups (at baseline), but this recovered to levels similar to Control in response to mitogen.

HDAC6 and HDAC11 were also increased in animals that only received milk from infected mothers. Wang et al.^24^ when using an HDAC6 inhibitor, observed a reduction in HDCA6 recruitment to the IL-10 promoter along with enhanced TNF-α, IL-12p40, and IFN-γ production, as well as increased influx of macrophages, dendritic cells (DCs), and neutrophils to the lungs in response to *Mycobacterium tuberculosis* infection. In fact, Cheng et al.^6^ reported that HDAC6 is required for transcriptional activation of IL-10 gene expression in macrophages and dendritic cells through STAT3 activation, while HDAC11 acts as an IL-10 transcriptional repressor. Here, the expression of HDAC6 seemed to overlap with the repressive effect of HDAC11. These data are corroborated by the increased IL-10 production in macrophages (CD14+IL-10+) in the SIM group. It is worth noting that the increase in HDAC6 expression, together with the baseline HDAC5 decrease in breastfed animals, may be related to the decrease in Treg cell activity and markers (FoxP3, CTLA-4 and GITR).^25^ ^,^ ^26^ These data are corroborated by the lower frequency of CD4+CD25+FoxP3+ cells in animals which were breastfed under mitogenic stimulus. Regarding the production of cytokines by T lymphocytes, decreased production of IL-4 and IFN-γ was observed (although there was no statistical difference for the latter). There was a subtle increase in the frequency of IL-10 producing B lymphocytes under mitogenic stimulation, and the baseline frequency was high in all study groups. These data corroborate the dependence of IL-10 on phagocytic cells in producing the suppressive and epigenetic effects of breast milk from schistosomotic mothers.

The high expression of HDAC10 was related to activation of the IL-1β-mediated NF-κβ signalling pathway in a study with mesenchymal stem cells derived from the synovial membrane in temporo-mandibular joint repair.^27^ However, HDAC10 was shown to be important in regulating the production of reactive oxygen species (ROS) in gastric cancer, and its inhibition led to the accumulation of ROS, triggering the intrinsic apoptotic pathway.^28^ It is known that ROS acts both in the innate and adaptive immune systems by attracting polymorpho-nuclear leukocytes, monocytes, and macrophages through Toll-like receptors (TLR).^29^ ^,^ ^30^ Here, there were increases in the expression of this enzyme in the group that received only milk (SIM) as well as in those that were born and breastfed (BSIM). This finding may be related to the activity of antioxidant enzymes in breast milk.^31^

Milk from infected mothers also led to increased expression of class III HDACs, Sirt2, and Sirt7. It is known that Sirt2 acts on the anti-inflammatory pathway in M2 macrophages through expression of *Arginase 1 (Arg1)* and *Cd11c*,^21^ and reduces ROS by increasing superoxide dismutase 2 (SOD2), catalase, and glutathione peroxidase.^8^ The increase in Sirt2 was related to inhibition of the NF-κβ pathway and reduced expression of IL-1β, IL-6, and TNF-α.^21^ Sirt7 acts on the stabilisation of TGF-β receptor type 1, allowing for efficient signalling of TGF-β.^32^ This targeting to an anti-inflammatory response profile is consistent with the tolerogenic effect of breast milk.^33^ According to our data, offspring who were only breastfed by schistosomotic mothers and who had undergone postnatal infection by *S. mansoni* also had elevated levels of TGF-β, in addition to decreased nitric oxide production (Unpublished observations).

High expression of Sirt6 was observed in all experimental groups (born and/or breastfed animals). According to Lee et al.,^20^ high expression of Sirt6 acts to inhibit the NF-κβ pathway and blocks the effect of TNF-α. Li et al.^34^ showed that overexpression of Sirt6 inhibits RIG-I-like receptor (RLR) and Toll-like receptor 3 (TLR3) in Dengue virus (DENV) infection, and the sirtuin core domain of SIRT6 is required for the inhibition of NF-κB p65 function, negatively regulating DENV-induced inflammatory responses via the RLR and TLR3 signalling pathways. These observations support the inhibition of inflammation we observed in the offspring of schistosomotic mothers.

The high expression of Sirt5 was also observed in the SIM group. It has been shown that Sirt5 is responsible for deacetylating STAT3, disrupting its activity at the mitochondrial level.^35^ Qin et al.^36^ demonstrated that deficiencyof this enzyme led to a decrease in the innate inflammatory response, with lowered production of IL-6, TNF-α and, MCP-1 both in the hyper inflammatory and hypo inflammatory stages of sepsis in an animal model. In addition, it was observed that high expression of Sirt5 leads to an increased pro-inflammatory response by decreasing the interaction between Sirt2 and NF-κβp56.^36^ Here, there was no decrease in Sirt2, and the increase in Sirt6 and Sirt7 may have overlapped with the effect of Sirt5.

Thus, HDACs were epigenetically altered by maternal infection and were involved in the suppression of pro-inflammatory mediators and increased IL-10 production, but did not favour cells with a regulatory phenotype. Even knowing the importance of cell-cell interactions in both *in vivo* and *in vitro* systems, it would be important to culture purified cells to understand if this pattern of expression of HDACs, indicating a predisposition to IL-10 dependent anti-inflammatory effects, was due to previous programming of T lymphocytes and macrophages in the BIM and SIM groups, respectively.

In conclusion, gestation in infected mice led to increased expression of HDAC9 alone, while breastfeeding from schistosomotic mothers led to increased expression of HDACs of all classes. It is true that the extrapolation of these data from mice to humans should be carefully evaluated, but our findings highlight the importance of experimental and clinical approaches to investigate the efficacy of therapeutic targets in allergy, autoimmunity, and cancer models after long-term epigenetic changes result in offspring from areas endemic for schistosomiasis. Therefore, our results shed light on immunological factors resulting from infection during early age, caused by modifications in epigenetic profile. In view of this, it will be important to evaluate factors in breast milk of infected mothers and how these components may modify the epigenetic and immunological profile of offspring.
